# The Circulation of Scientific Articles in the Sphere of Web-Based Media: Citation Practices, Communities of Interests and Local Ties

**DOI:** 10.1371/journal.pone.0158393

**Published:** 2016-07-28

**Authors:** Muriel Lefebvre, Julie Renard

**Affiliations:** LERASS, Université de Toulouse, Toulouse, France; Universidad de Las Palmas de Gran Canaria, SPAIN

## Abstract

On 5^th^ December 2012, a scientific article reviewing a change in the feeding behaviour of the European catfish, one of the largest freshwater fish, was published in the American scientific journal, *PLOS ONE*, an open access journal, which also allows the mass publication of pictures and videos. Within a few days following the publication of this article, it was relayed by numerous web sites and generated a media craze. In this paper, we analyse the circulation of this scientific information in the sphere of Web-based media during the two months following its publication, by revealing the citation mechanisms of the original article and the logic of the Internet users participating in its diffusion. In addition, since the circulation of its informational content travelled beyond linguistic and geographical boundaries, we chose to compare the citation modalities and intertextual relationships of documents in the three countries where the article spread the most widely, namely: France, the United States and Great Britain. Even though our study shows that the media circulation of scientific papers operates in a traditional way, the intertextual analysis underlines the grand variety of participants (such as journalists, non-scientists, fishermen, technology enthusiasts and Internet users) involved in the diffusion of this information, each of them mobilizing different intertextual strategies, according to their various targets. They all transformed, reformulated and appropriated the scientific information according to their own, unique interests. This study also emphasizes the importance of journalistic websites as opinion relays. They were the first diffusers involved in spreading the information but this role was rarely acknowledged by the Internet users - through citations, for example. In contrast, we observed that amateurs’ communities (communities of practices and communities of interest of fishermen or of buzz fans), which only became involved in a second temporal phase of the spreading, preferred to build up their credibility through citations of the original article. Finally, this research helps to rethink the mechanisms of the circulation of scientific information in the Web-based media, highlighting both the variety and the inventiveness of the interactions between the academic and public spheres.

## Introduction

On 5^th^ December 2012, a scientific article revealing a change in the feeding habits of the European catfish, the third largest freshwater fish, was published in the American scientific, open access journal, *PLOS ONE*[1]. Researchers observed catfishes “beaching” out of the water to catch pigeons on the bank. Within a few days, this information was relayed by numerous websites and generated a real media craze. The original article and the associated video showing several attempts of the catfish to catch pigeons were viewed more than four million times, with downloads and online comments [2], on a wide variety of Internet media sites – those offered by traditional media (newspapers, radio, television), infomediaries (news portals and search engines), native sites (participative sites), blogs (independent or linked to other sites) and numerous institutional and associative websites (fishermen's associations, university, etc.). This variety of websites reveals the wide diversity of online communities and Internet users that chose media relays to circulate this information.

This paper meticulously analyses the circulation of this scientific information in Web-based media during the two months following its publication.

The research is based on science studies and on media studies. More precisely, through a content analysis of an appropriate corpus, we study how such scientific information circulates independently of its original sphere of publication, quite often without any “obvious” links (i.e. without any references) to the original article, the source journal or even the scientific institution. This study thus gives a new perspective on the interactions between experts (the researchers, writers of the original scientific article), journalists and amateurs.

A cartography of the intertextual links among all the websites considered points out the temporal and the social dimension of how the information spread. From a temporal point of view, this study shows that such spreading followed exactly the same modalities as media information. However, from a social point of view, the study underlines the different parties involved, each of them mobilizing different intertextual strategies, according to their various targets. Our results emphasize the importance of journalistic websites as opinion relays - they were the first diffusers involved in the spreading - whereas communities of amateurs (communities of fisherman, communities of buzz fans, etc.), which are distinct from traditional media diffusion, only became involved in a second temporal phase. We finally show that these communities of practices and of interest appropriated the scientific information coming from the original article differently, in terms of temporality, of geographical surroundings, of citation modalities and, above all, of content (behavioural change in the European catfish feeding habits versus sensational information).

Since this information circulated beyond linguistic and geographical boundaries, we chose a qualitative methodology to compare citation modalities and intertextual relationships of documents in the three countries where the article had the largest diffusion: France, the United States and Great Britain.

### When the study of scientific citations takes an interest in new media mechanisms

The networks of academic and scientific citations have been the subject of many studies since the 1960s and are now very well analysed and understood by bibliometric tools, which have been designed to observe the production and circulation of scientific information in the academic sphere ([3], [4], [5], [6], [7], etc.) However, these bibliometric studies focus on traditional forms of scientific publications, such as scientific magazines, scientific literature, scientific patents and scientific conference proceedings, while the development of the Internet and, more recently, the evolution of the social networks, have led to modifications of the landscape of scientific publishing and, therefore, their citation modalities.

Thus, whether it is through open archives or electronic free access journals [8], different devices and alternatives to bibliometric indicators have been developed in order to evaluate scientific research. For example, Almetrics tools count the number of times an article has been downloaded and also the number of times it has been quoted, on Twitter or Facebook for instance. These alternative metrics are used to measure the visibility of an article on the social networks [9][10], by considering "pure readers" in particular, i.e. researchers and attentive readers, some of whom do not necessarily write research papers but may quote them on Twitter or scientific blogs. Scientific blogs run by researchers are also expanding rapidly [11]. Several studies have focused on the citation networks among scientific blogs or between blogs and academic publications using bibliometric tools [12]. For example, Luzon [13] has highlighted the various factors leading to the citation of references in a blog, and notably the strategic use of these links by academic bloggers "*to seek their place in a disciplinary community*, *to engage in hypertext conversations for collaborative construction of knowledge*, *to organize information in the blog*, *to publicize their research*, *to enhance the blog’s visibility*, *and to optimize blog entries and the blog itself*" (2008). He concluded that the hypertextual links in scientific blogs are used for the purposes of rhetoric, to increase the value of a blog and to contribute to its publicity.

Furthermore, research focusing on the citation of academic articles by other networks than those of specialized publishing is a particularly fruitful field of investigation. Nevertheless, whether we are talking about academic social networks, scientific blogs or social websites, citation analysis must utilize a suitable methodology. Online citations do not follow the rules of academic citations, either in terms of practical modalities or in intentions. Hence, a specific approach and analysis is needed.

### Online citations by non scientists, beyond the academic world

This paper analyses citations of a publication beyond the academic sphere. We studied the circulation of information and citations in the Web-based media and specifically *PLOS ONE*, an open access journal, the contents of which are freely accessible from any device connected to the Internet.

We based our study on two theoretical frameworks: (i) the theory concerning the circulation of information in the media and (ii) the dissemination models concerning scientific knowledge. The models proposed by these two theoretical frameworks are related to one another, “*Science coverage in the mass media was and still remains the major channel that bridges the gap between science and the general public*. *Most people*, *including many decision-makers*, *acquire their information about science mainly*, *or even exclusively*, *from the mass medias*” [14].

#### Theories of the circulation of information in the media

Many theories attempt to establish a model of the flow of information in, and its circulation via, the media. Here, we only describe those that are the most relevant for our study. For instance, Katz and Lazarsfeld’s two-step flow theory [15], developed in the 1970s, questioned the effects of media on the public and the role of opinion leaders (e.g. how those who relay the information can indirectly influence individuals). This approach remains a reference in many areas, such as marketing, and is also very relevant in studies on the diffusion of innovations [16]. Undeniably, the notion of "opinion leader" is effective in describing the logic of influence between the various participants. Moreover, the “Uses and gratifications” model questions users’ motivation and reveals the communicational value of the information exchanged. This theory is also mobilized in knowledge management research [17] in order to identify the motivations behind the sharing of information on academic forums (e.g. information sharing, problem solving, debates, shared interests, etc.). In the present study, this concept allowed us to understand the motivations of Internet users to cite a research article. However, the analysis of the social meaning of the motivational actions to share information have often been sidelined in this model, which has led research in cultural studies to further contextualize media usage by taking the variety of profiles and life experiences into account, and considering the general public as participants in their own history.

More recently, research on mediacultures or on the convergence culture [18] has fostered a new understanding of the links among media, the general public and culture by showing how participants can build a specific form of reality through media mediation.

#### Theories of the ‘dissemination’ of knowledge

Scientific studies have theorized ideas of knowledge circulation mechanisms in the media based on three main paradigms [19] [20] [21] which are directly related to the media theories previously discussed. The oldest model is the "lack of scientific knowledge by the public". This top-down linear model, which prevailed in the 1970s and 80s, considers the general public as a relatively passive homogeneous whole. Later, in the 1980s, in the prevalent context of questioning scientific authority and of public scientific controversies, a model "of dialogue" emerged, urging its users to take the various participants in the sphere of sciences into account. Since then, the participatory model developed in 2000 has considered science as an area of coproduction of knowledge, where knowledge amateurs intermingle with knowledge experts [20]. This third model is based on the renewed vision of knowledge production modalities developed by Nowotny & al. [22]. According to their descriptions, the two modes of knowledge production are in opposition: Mode 1 describes science as governed by the academic interests of a specific community and Mode 2 is characterized by collaboration not only between different scientific disciplines but also between scientific communities and the socio-economic world. In fact, in the example analysed here, it is important to point out that it was the fishermen who alerted researchers to the change in catfish feeding habits. This third model, the so-called "participatory culture", is a very similar to the one developed by Jenkins [18] on the culture of convergence, which unites multiple participants and media in a collaborative approach. It is based on Web 2.0 features, which draw on the media to broaden the audience for specialized content. Today, there can be no doubt that the general public has "access to information and resources previously accessible only for journalists, for enlightened amateurs, or for researchers” [23].

Nonetheless, it is essential to emphasize (c.f. Bensaude-Vincent [19]) that these knowledge dissemination regimes now coexist and are not mutually exclusive. In this paper, we show that their coexistence reflects an emerging situation of knowledge circulation, appropriation and creation based on the analysis of the circulation of a scientific article in the media.

## Materials and Methods

The analysis of scientific citation practices in research is usually based on well-identified bibliometric indicators but no such protocol exists for the study of media citation modalities. Therefore, we designed a specific methodology to analyse the circulation of scientific information in the Web-based media sphere, which took all intertextual traces into account at the same time – in this case, references to the original article in *PLOS ONE* (standard academic reference) and citation of a part of the original article (whether textual, visual - photos or drawings, or video).

First, we performed a search on Google to identify intertextual traces of the original article on the Internet websites. Then, we manually extracted the data relevant to our research: quotes and links (to *PLOS ONE* journal, to the authors’ names or to the scientific institutions involved), presence of photos or videos coming from the original article, links to other important articles, release date, topic, theme, etc. In order to obtain a homogeneous corpus, we suppressed all the articles written before 5^th^ December 2012, or clearly not related to our topic (recipes for cooking pigeons, etc.).

Secondly, for the French corpus, we observed that, although some items referred to the original article in *PLOS ONE* or to other important websites, this was not always the case. So, we conducted a content analysis, through different indicators (use of common statistics, use of common sentences, use of hypertextual links) to map the circulation and the citation mechanisms of the original article.

### Census of the websites evoking the change in Catfish feeding behaviour

To start, we decided not to consider the social networks online, such as Facebook or Twitter, because of their specific diffusion mechanisms [9][10]. Our study focused on non-academic websites reaching a broader audience than the academic one. Non-academic websites diffusing this information were identified using the Google search engine on 31^st^ December 2012 with the following key words: "*silure et pigeon*" for France, and "*catfish and pigeon*" for the United States and Great Britain. The choice of Google, a non-specialized, popular search engine, reflected our desire to identify as many of the non-academic websites as possible and to identify the way this specific content could circulate, sometimes without an obvious link to the original information. One of our goals was to identify links other than those occurring through clear references (to the authors, to *PLOS ONE*, or to the scientific institutions concerned).

The number of results was large enough for us to limit our corpus to the first fifty pages of the Google search results. After suppression of the academic results, of articles written before 5^th^ December 2012 and of articles clearly not related to our topic (cookery recipes, etc.), we identified 210 posts unevenly distributed among the three countries: 128 in France, 49 in the United States and 33 in Great Britain. Even though the number of articles differed from country to country, the in-depth content of our corpus allowed some major trends to be identified.

Additionally, the research highlighted the methodological issue raised by the attempt to carry out systematic analysis of information pathways on the Internet. Types, nature of media, status of authors and readers were numerous and tightly interconnected. All attempts to classify websites strictly according to their type (such as media status for instance) failed, as a given piece of information often possessed more than one status: The *Passeur de sciences* Blog is both Pierre Barthélémy’s blog and strongly affiliated to the French newspaper *Le Monde* as it is hosted by the paper’s website. However, it was still necessary to analyse the information items, so a new way to analyse the information flows and pathways online had to be found.

#### Ranking by media type

We ranked the sites relaying the information by media category and pooled the data in an analytical table.

Six categories were defined:

- **Online version of traditional media (VL)**: *La Dépêche* (Fr), *France Inter* (Fr), *The Week* (US), *The Blaze* (US), *The connexion* (GB), *Birdwatch* (GB), etc.
- **Sites Native to the Web** (NW, information sites existing only online): *Actuzz* (Fr), *Carré d’info*, *Fun-buzz* (Fr), *Business insider* (US), *Grist* (US), *Mail Online* (GB), *Descrier* (GB), etc.
- **Content Aggregators**: *NewsYahoo* (Fr), *Google actualités* (Fr), *Geekosystem* (US), *Yahoo News* (US), *Travel AOL* (GB), *Noxxed* (GB), etc.
- **Forums**: *SilureGlanis* (Fr), *Carpe Alsace* (Fr), *io9* (US), *Carp Forum* (GB), *Fishkeeping* (GB), etc.
- **Blogs**: *2tout2rien* (Fr), *Littlefishing*, *America Blog* (US), *ZME Science* (US), *Pigeon Mania* (GB), *Following the nerd* (GB), etc.
- **Sites** (administrations, corporations, associations): *Terra Femina* (Fr), *Amis des Amis* (Fr), *10000 Birds* (US), *GeekyDump* (US), *Aqcenter* (GB), *Grindtv (*GB), etc.

S1 Fig shows the proportion of categories of each site in each country.

Boundaries between the chosen categories are complex and porous. The choice of these categories nevertheless proved to be useful for analysing the circulation of the *PLOS ONE* article in the Internet media sphere. Each type of site has, by its nature, a specific editorial line, a different editorial status engaged (the expert-researcher, the journalist or the amateur), distinct reader agreements [24] and various ways to appropriate and process information.

#### Ranking by theme

To complete this analytical table, we designed a thematic classification to analyse the editorial treatment of the *PLOS ONE* article citations. Depending on the site, we observed two distinct editorial choices: in the first case, the change in the catfish feeding behaviour was highlighted while, in the second case, the oddness of the information or even problematic fishing policies were underlined.

Seven themes were identified, some of which were broken down into the following sub-themes:

- **General information (News):** 2*0min* (FR), *Canal plus* (FR), *Fark*.*com* (US), *Grist* (US), *The connexion* (GB), *French News Online* (GB), etc.
- **Media and Technology (M&T)**: *Actuzz* (FR), *Divertissonsnous* (FR), *Reviewer* (US), *Geekosystem* (US), *Business Insider* (US), *Gismodo* (GB), *Digital Spy* (GB), etc.
- **Fauna and Flora (F&F)**: *La pêche et les poissons* (FR), *Actualités news environnement* (FR), *Animal Fact guide* (US), *LiveLeak* (US), *Fishkeeping* (GB), *Pigeon Mania* (GB), etc.
- **Science and Technology (S&T)**: *Sciencesetavenir* (FR), *Futura-Sciences* (FR), *Discover* (US), *SciTech Daily* (US), *etc*.
- **Odd and Funny (H& I)**: *Mortderire* (FR), *vidéo-buzz* (FR), *Blog's avenue* (US), *Tobefun* (US), *Boreme* (GB), *Anorak* (US), *etc*.
- **Academic:** *Paul Sabatier University* (FR), *CNRS* (FR), *Smithsonian (US)*, *etc*.
- **Diverse**: (category that includes disconnected themes such as rural life, the paranormal, health and individual initiatives): *Nous ne sommes pas seuls* (FR), *Fédération des acteurs ruraux* (FR), *EarthTouch TV* (US), *Grindtv* (GB), *etc*.

Some themes or types of sites maintained rather vague borders (S2 Fig). For example, some media were not present in the whole corpus. This was the case for academic sites such as the CNRS or University Paul Sabatier, for which there are no equivalents in the United States or Great Britain. Likewise for the “Science and Technology” theme, which was not represented in Great Britain.

This element led us to develop thematic categories not from the way the contributors themselves defined the themes of their websites, but rather through a content analysis of our corpus websites.

### Qualitative content analysis methodology

The corpus disparity among the three countries analysed and the geographical anchorage of the contents of the scientific article led us to treat US, British and French documents differently. On the basis of the variety of information we found, as explained above, we developed a comparative analysis of the circulation of the original information in all three countries, exploring the date of publication, the previously defined categories and, finally, the number of sites citing the original article. Then, we conducted a qualitative analysis of the French corpus, involving content analysis tools with a specific focal point. Three different indicators were considered in this intertextual analysis: use of common statistics, use of common sentences, and use of hypertextual links. This study helped us to map the circulation and the citation mechanisms of the original article.

## Results

The circulation of the content from the original article and its relay in the Web-based media did not, in itself, specifically relate to its scientific dimension. It operated in a very similar scheme to the framework of the two-step flow theory [15]. The citation modalities, as described above, concerning the *PLOS ONE* article in the public sphere were, on the other hand, quite surprising and can be referred rather to community practices with an informational reappropriation mechanism. The analysis of this "participatory culture" as defined by Jenkins [18] demonstrated the involvement of Internet users in the circulation, the reformulation and the processing of contents.

### Temporal circulation of information

We first analysed the temporal circulation of information in the 210 international media platforms identified in each country. S3 Fig shows the relationship between the number of items and their webcast date in the three geographic areas of our corpus.

The essential part of the diffusion took place in the week following the publication of the article by F. Santoul and his team, on 5^th^ December 2012. The diffusion followed a bell curve: it grew extremely quickly and then decreased just as rapidly.

In the United States, 94% of the articles posted appeared before 13^th^ December 2012 (only 3 of them were after that date). The same pattern was observed in France for 89% of the articles analysed (114 of the 128 articles analysed were published before mid-December). Finally, in Britain, we observed a milder version of the same diffusion phenomenon with 75% of the articles analysed (25/33) posted within a week of the first publication in *PLOS ONE*.

Despite the difference in size of the corpus, we found that the temporality of article circulation was almost identical from one country to another: its mid-life was 4 days in all cases. The number of articles relaying this information decreased from 9^th^ December. This relatively brief and intense event which then fizzles out rapidly is a characteristic phenomenon of a "media buzz" [25].

### Range of citation modalities: the analysis of intertextual relationships

The attempt to understand the temporal circulation led us to analyse the participants involved in the diffusion of the scientific information. We did this through an analysis of the content underlying the intertextual citation and reference mechanisms between the various websites studied.

#### Key role of leading sites, true diffusion nodes

In analysing the circulation modalities of the article on the European catfish, we quickly identified the leading sites, the “real nodes of diffusion” in the three countries of our study. Similarly to what the *two-step flow theory of communication* identifies as "opinion leaders," these "source" documents - true "opinion formers" - seemed to emerge as unavoidable diffusion nodes on the Web. These leading sites were more than just relays: they played an advisory role in advertising content worthy of being webcast by others.

The main US source was the blog, *Not Exactly Rocket Science*, (webcast of 5^th^ December) by Ed Yong, a British science journalist for the American promotional magazine, *Discover*, (26.5% of the US articles analysed cited this source).

In Great Britain, 15% of the analysed records cited the *Daily Mail* (webcast of 7^th^ December), an online version of the British newspaper, while only 9% mentioned Ed Yong’s blog.

In France, the main opinion leader’s website was the *Passeur de Sciences* (PDS) blog (webcast of 6^th^ December), run by Pierre Barthélémy, a science journalist for *Le Monde*, a daily newspaper (34.5% of French documents referred to it). Just like "pure scholar readers" [10], "journalist bloggers" turn away from their usual practices to write promotional articles and, as a result, participate in the circulation of scientific information. These blogs, run by scientific journalists, play a major role in the dissemination of scientific knowledge.

On 7^th^ December, two other sources played a key role in France: an article from *La Dépêche du Midi (LD)*, a regional daily newspaper (20.5% of the documents referred to it) and the *Agence France Presse* (*AFP)*, a world news agency, (19.2% of documents referred to it).

This first analysis shows that the principal information relays, whether in France, the United States or Britain, are journalistic sources (such as articles in the online media and journalists’ blogs). Today, journalistic sources remain references, even on the Web. Gradually, the information moved further into the amateur sphere [19] [20], through other participants and other types of media support (blogs, association sites, etc.).

Furthermore, in France, citation analysis of the documents identified showed that most of them repeated the information webcasted by the three nodes mentioned above but without making explicit reference to them. It was on the basis of "key words", numbers, geographical indicators and extracts of interviews of F. Santoul that we identified the intertextual relationships between these different documents.

Likewise, only 20 French sites cited *Passeur de Sciences* as a source when, in fact, 26 of them used the same wording. For the *AFP*, the gap was even larger as only 3 out of the 15 identified articles explicitly referred to the source. What was more important was the dissemination of the content and not the secondary source that attracted attention to the same content.

S4 Fig below gives a clear account of the complexity of the circulation network of the *PLOS ONE* article in the Web-based media sphere and highlights one of the relay nodes analysed in France: the scientific blog *Passeur de Sciences*.

The identification of this singular content in the various relays allowed us to reveal the intertextual phenomenon occurring on the Internet in a broader way. References and citations did circulate from one site to another; Internet users modified and appropriated the content that they, in turn, published. The *Passeur de Sciences* case was not isolated since it appeared for each of our three main opinion leaders in France. This phenomenon corroborated the role of hub-sites as opinion leaders playing an implicit role as advisors of mediatized contents. The fact that the leading sites took time to mention a certain piece of information eventually led many "trend-setting" users, usually members of diverse communities of interest, to take over this information. By doing so, these “trend-setting” users seemed to aim more at publicly displaying their membership of the digital sphere and social networks than at contributing to the visibility and recognition of leader sites (through references or citations).

#### Academic references

This dissemination was duly sourced from an academic point of view and, from this perspective, we are coming closer to understanding the circulation modalities of scientific information in academic blogs [13]. References to the original article in *PLOS ONE* were present in almost half of the articles analysed in the three countries. Consequently, the article from *PLOS ONE* was cited or mentioned through a link in 46.9% of the websites in the US, 35.1% of the websites in France and 30.3% of the websites in Great Britain (S5 Fig).

Institutions (laboratories or universities) were slightly less referenced but in similar proportions: 40.8% of the US sites cited institutions versus 27.3% of the French sites and 24% of the British sites.

This phenomenon may seem surprising, since, on the one hand, the relaying sites were rarely mentioned but, on the other hand, the analysed sites maintained no explicit link to the academic world. Therefore, the citation and referencing codes in this realm were not supposed to be known by the Internet users. However, the authors of the identified documents had obviously made the effort to refer to the original article in their own published article. But what were the intentions of these authors? Was it an ethical concern or a rhetorical strategy to support their views and demonstrate their knowledge of scientific codes, and hence their membership of academia? Luzon [13] has shown, in the case of scientific blogs, how academic references and citations were identified as increasing not only the value of the blog but also the credibility of the author *vis-à-vis* a community of interests (a community of individuals sharing common interests) [26]. A similar phenomenon was observed in our study: the reference to the original article, clearly identified as coming from the established academic world, increased the credibility of the websites in question.

Nonetheless, there was a fundamental difference between the three countries: even though the *PLOS ONE* article was co-written by six authors, they were not cited in the same way in the documents analysed. Their rank in the list of authors was decisive, as in the bibliometric analysis of scientific publications. However, R.K Merton stated, "As we examine the experiences reported by eminent scientists we find that this pattern of recognition, skewed in favour of the established scientist, appears principally (i) in case if collaboration and (ii) in cases of independent multiple discoveries made by scientists of distinctly different rank." [27]. Only two authors out of six were cited here: the first on the list of authors, Julien Cucherousset, who did the experimental work, data analysis and co-editing, and Frédéric Santoul, last author on the list, who was the scientific director of the research project, identified as having participated in all phases of the research from experimentation to writing the article. Depending on the country from which the articles came, the editors did not refer to the same researchers. In the United States and in Britain, Julien Cucherousset was the representative scientist whereas, in France, the most often cited researcher was Frédéric Santoul. These two different treatments can be partly explained by the origin of the sources that the contributors used or, as demonstrated by Merton [27], by the necessity to highlight Julien Cucherousset, since his status was directly linked to the financing of the catfish study (Julien Cucherousset received an “ERG Marie Curie” grant (PERG08-GA-2010- 276969) in the EDB laboratory, part of the “Laboratoire d”Excellence” (LABEX) named TULIP (ANR -10-LABX-41)).

In the American and British network, Ed Yong and the *Daily Mail* cited Julien Cucherousset as the first co-author, reflecting the specific citation modalities of the academic world. In contrast, in France, Frédéric Santoul was the only author cited (23.4% of the sites analysed). As a team leader, he was interviewed by two of the three main diffusion nodes identified above (*Passeur de Sciences* and *La Dépêche*). From then on, his name was quoted almost systematically by the other websites, providing this source with greater legitimacy each time. This researcher quickly became emblematic of the research on European Catfish in France. The concepts of source and copyright experienced significant changes in terms of the relays identified. While circulating, information became loaded with new embedded values put forward by the different participants who contributed to its circulation.

#### Citing photos and videos

The audiovisual material also played a decisive role in the intertextual relations between the original article in *PLOS ONE* and the subsequent Internet postings. The role of images and videos, used as eye-catchers in the dissemination of scientific blogs, has notably been highlighted by Ranger & Bultitude [28]. In our case, the media scrutinized were especially relevant to this phenomenon: the video posted by *PLOS ONE*, followed by YouTube, flourished internationally on the Web. On S6 Fig, we can see that this video was present in 89.7% of the American websites studied, in 69.5% of the French websites and 66.6% of the British websites. The Internet postings did not necessarily refer to the original video link on *PLOS Media* but rather cited a link to a video posted several times on YouTube by Internet users. The spectacular nature of this video was an essential element in explaining this extensive diffusion and circulation [29].

On the other hand, the photos picturing the significant difference in size between the pigeons and the catfish and showcasing competition between two natural environments were much less posted: 38.7% of the US websites used them versus 29.6% of the French websites and 24.2% of the British websites. The use of these photos and videos played a central role in the circulation of the reference of the original article, even if they served to show the spectacular nature of the phenomenon rather than the change in the European catfish feeding habits. Yet, beyond their eye-catching nature (or maybe because of it?) these videos and images played the content accreditation role in very much the same way as the references to the original article did.

Furthermore, some native websites, blogs and forums highlighted different photos from the ones provided by *PLOS ONE* or by the key site–notes identified above. In these articles, this difference from the original article was due to the authors’ background - often curious individuals, pigeon fanciers or fishermen. Although posting personal fishing photos (often of the fisherman’s own catch) is common in France (7.8% of the websites analysed), we also observed pigeons pictured as doves in Great Britain, which could have had a particular effect on the recipients of this information. The utilization and the staging of these photos and videos also reflected a particular appropriation of the content by the users. Depending on their target audience, contributors may divert visual symbols to raise awareness to their cause, e.g. pigeon preservation.

### Communities of practices and communities of interests

The study of the temporal and the social circulation of the scientific content from the original article led us to consider the context of uses: why would so many different digital users refer to this information?

S3 Fig, on the temporal circulation of information, shows that, from Sunday 9th December onwards, there was a significant drop in the number of websites citing the European catfish and a decrease in the diversity of the themes mentioned. This reduction of editorial activity mainly occurred among journalists on the Web and it was not the case for amateurs (who generally take over at weekends) as we noted large amounts of content posted in forums and on personal websites or blogs. This content was rehashed by online communities, which are structured around common practices or interests. These are the so-called “communities of practices” that were analysed by Wenger [26].

#### Diverse communities of practices and interests

The "Fauna and Flora" theme appeared repeatedly in France and Britain, followed by the fishing community, who seized upon the information. The "Fauna and Flora" section was fairly representative of the link between types of sites and themes. In fact, whether in France or Britain, the percentage of forums was relatively high compared to other types of media (22% and 27.2% of the web sites analysed). Most of these forums were devoted to fishing (57% of the forums in France and 66.6% in Britain) [30]. The fishing community, understood here as a singular community of interests and practices, was largely mobilized in both countries. The information was of interest to them not only through its ecological nature but also for its spectacular elements.

Conversely, this section was under represented and even marginal in the United States and consequently represented a small proportion of forum documents in our corpus (only 2% of the American websites analysed). In this case, fishermen were not particularly interested in the information since catfish are quite unusual in American waters.

Nevertheless, fishermen were not the only community to be interested in the science of catfish or to appropriate the original *PLOS ONE* article through information poaching and alteration processes [31]. Communities of interests as diverse as technology amateurs or online entertainment fans seized this scientific information and appropriated it through discursive regimes often very close to information spectacularization.

All of these communities played a specific role in the diffusion of this information on the web and participated in the media hype that this first series of documents generated. We specifically studied this feature in France. As shown in the chart below (S7 Fig), 21% of the French articles did not mention the behavioural change in catfish feeding but rather discussed the hype generated by this information on the Web. The buzz dimension appeared very early in the circulation of the *PLOS ONE* article. Importantly, it was not in the "Odd and funny" nor "Media and technology" sections but in two rather journalistic themes: "News" and "Fauna and Flora". In fact, it was mainly the journalists and the fishing community that contributed to the circulation and the media boost of the spectacular dimension of this information.

#### Geographical anchorage of communities of practices and communities of interests

The communities of practices and interests were also often communities anchored within a specific geographical territory. The research was conducted by a team of French researchers from the Midi-Pyrenees Region, located in the South West of France. Moreover, the catfish story was also rooted in that specific area and the scientific observations of its occurrence took place in that same region. Finally, many participants in the media, the scientific and the associative spheres were also from the same geographical area.

This phenomenon was rapidly identified in the leading article published by the regional daily newspaper, *La Depeche du Midi*. It stated the characteristics linked to the geographical anchorage of the research and its particular knowledge of that area. Hence, the characteristics of the place where the research was conducted were specifically mentioned: primarily the river Tarn but also Albi or its Cathedral (the small town of Albi, listed as a UNESCO World Heritage site, is locally renowned for its Cathedral) and it included finer idiosyncratic details like "the teacher and his students are photographed on the parapet of the Old Bridge, their eyes fixed on the island located below the dam". Finally, local specificities, such as the name of the university in Toulouse, also reinforced the local dimensions of the information.

More generally, this territorial anchorage reflected the proximity and the regular exchanges between the different people and social categories behind the circulation of this information: researchers, journalists and fishermen, were all guarantors of the disseminated information. This was demonstrated not only in the interview of the iconic scientist, Frédéric Santoul, who leads the research on catfish in France (key interview sentences were repeated on numerous occasions by many Internet users who, de facto, appropriated the researcher’s words without referring to the original article) but also in an interview with Stéphane Cabanes, the president of the local angling association.

The presence of visible local references, such as geographical statements or interviews of locally influential personalities, was rarely used in the American and British corpus we analysed; obviously these references to local peculiarities were not relevant for the audience abroad. This shows that a theme is more developed when it can be anchored not only in communities from a common area but also in local communities of interests. These two factors explain why the *PLOS ONE* article mostly circulated in France.

## Discussion

In this paper, we have updated the analysis of the circulation and citation modalities of a scientific article outside the academic sphere in a high visibility situation via the Web (most scientific articles will never be as commented as the *PLOS ONE* article analysed here).

Although our study shows that the media circulation of scientific papers operates in a traditional way, the intertextual analysis underlines the great variety of participants involved in the diffusion of this information, each of them mobilizing different intertextual strategies, according to their various targets. Journalists, non-scientists, fishermen, technology enthusiasts and Internet users all transformed, reformulated and appropriated the scientific information according to their specific interest. Thus, only a small minority of these Internet users referred to the scientific content of the original article (change in the European catfish feeding behaviour). The majority of them preferred the sensational dimension of the photos and the videos.

This study emphasizes the importance of journalistic websites as opinion relays - they are the first to be involved in the dissemination. Yet this role is rarely acknowledged by the Internet users, through citations, for example. In contrast, we observed that communities of amateurs (communities of practices and communities of interest of fishermen or of buzz fans), which only became involved in a second temporal phase of the spread of information from the article, preferred to strengthen their credibility through citations of the original article (sometimes with a hypertextual link to the original journal, *PLOS ONE*), of the authors, of the scientific institutions involved and, above all, of the amazing photos and videos accompanying the original article.

This point underlines an original way of spreading scientific information, which is distinct from the traditional media mechanisms and very close to the convergence culture described by Jenkins [18]. Amateurs are coming closer and closer to the academic source.

Finally, this research helped to rethink the mechanisms of the circulation of scientific information in the sphere of Web-based media, highlighting both varied and inventive interactions between the academic and public spheres. It would be interesting, in this regard, to pursue this work with a study on citation modalities in the social networks with a tool such as *Almetrics*. This would allow an in-depth study of the exchange dynamics at work in a borderline area where experts and amateurs intermingle.

## Supporting Information

S1 Fig Breakdown by type of site and by country. (TIF)

S2 Fig Breakdown by themes and country. (TIF)

S3 Fig Temporal circulation of information in the international media platforms identified. (TIF)

S4 Fig Circulation network of the scientific article in France. This figure gives a clear account of the complexity of the circulation network of the Plos One article in the Web-based media sphere and highlights one of the relay nodes analysed in France: the scientific blog Passeur de Sciences (Le Monde).
(TIF)

S5 Fig Percentage of quotes and links in each country. (TIF)

S6 Fig Citing photos and videos. (TIF)

S7 Fig France: proportion of articles in the "media buzz" by categories and publication dates. (TIF)

S1 File List of the websites analysed. (XLSX)
