# Supplementary figures and images for: The Circulation of Scientific Articles in the Sphere of Web-Based Media: Citation Practices, Communities of Interests and Local Ties
Source: PLoS One. 2016 Jul 28;11(7):e0158393. doi: 10.1371/journal.pone.0158393 (PMC4965155; doi:10.1371/journal.pone.0158393)

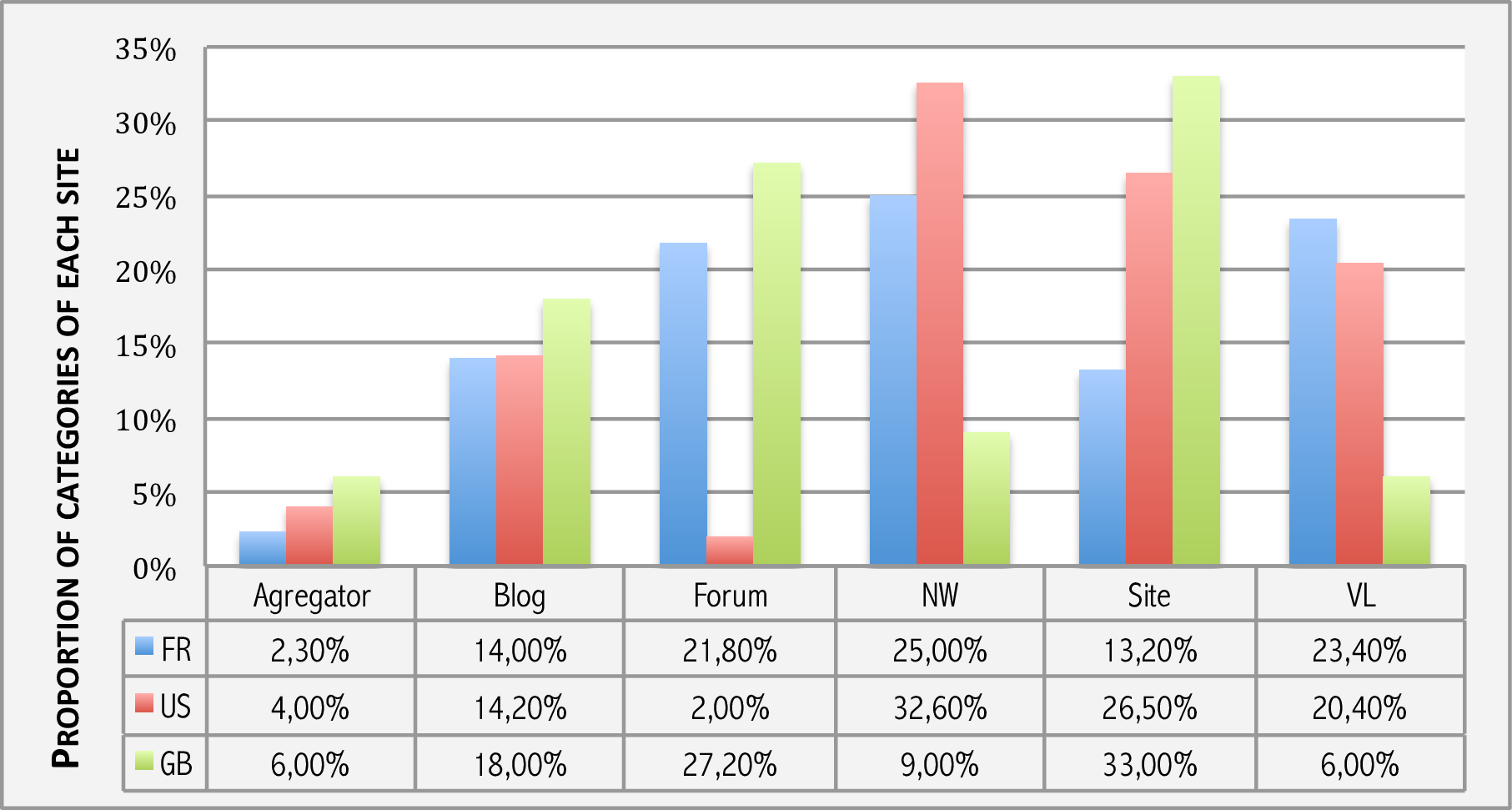

Supplement: S1 Fig — (TIF) [file pone.0158393.s001.tif]

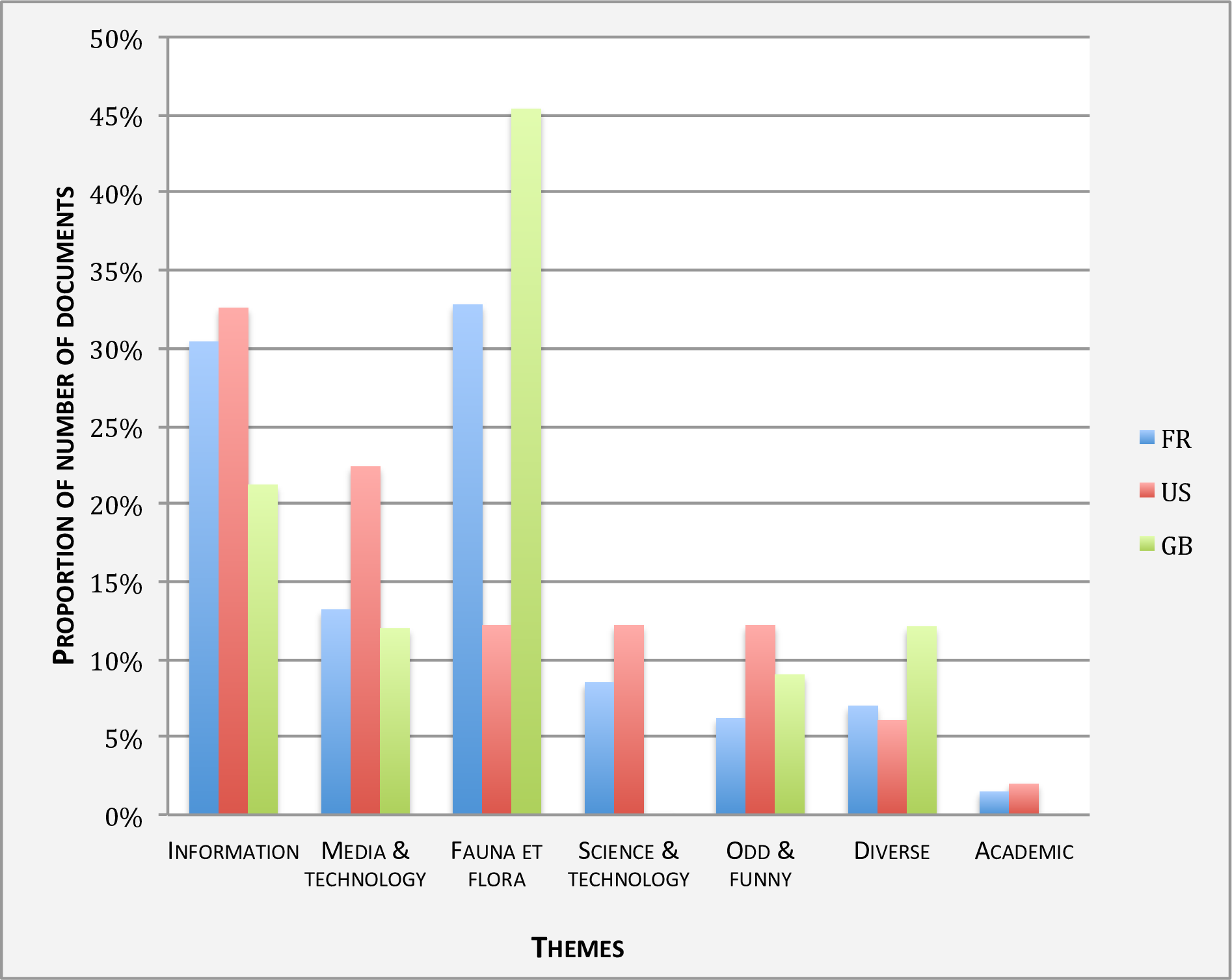

Supplement: S2 Fig — (TIF) [file pone.0158393.s002.tif]

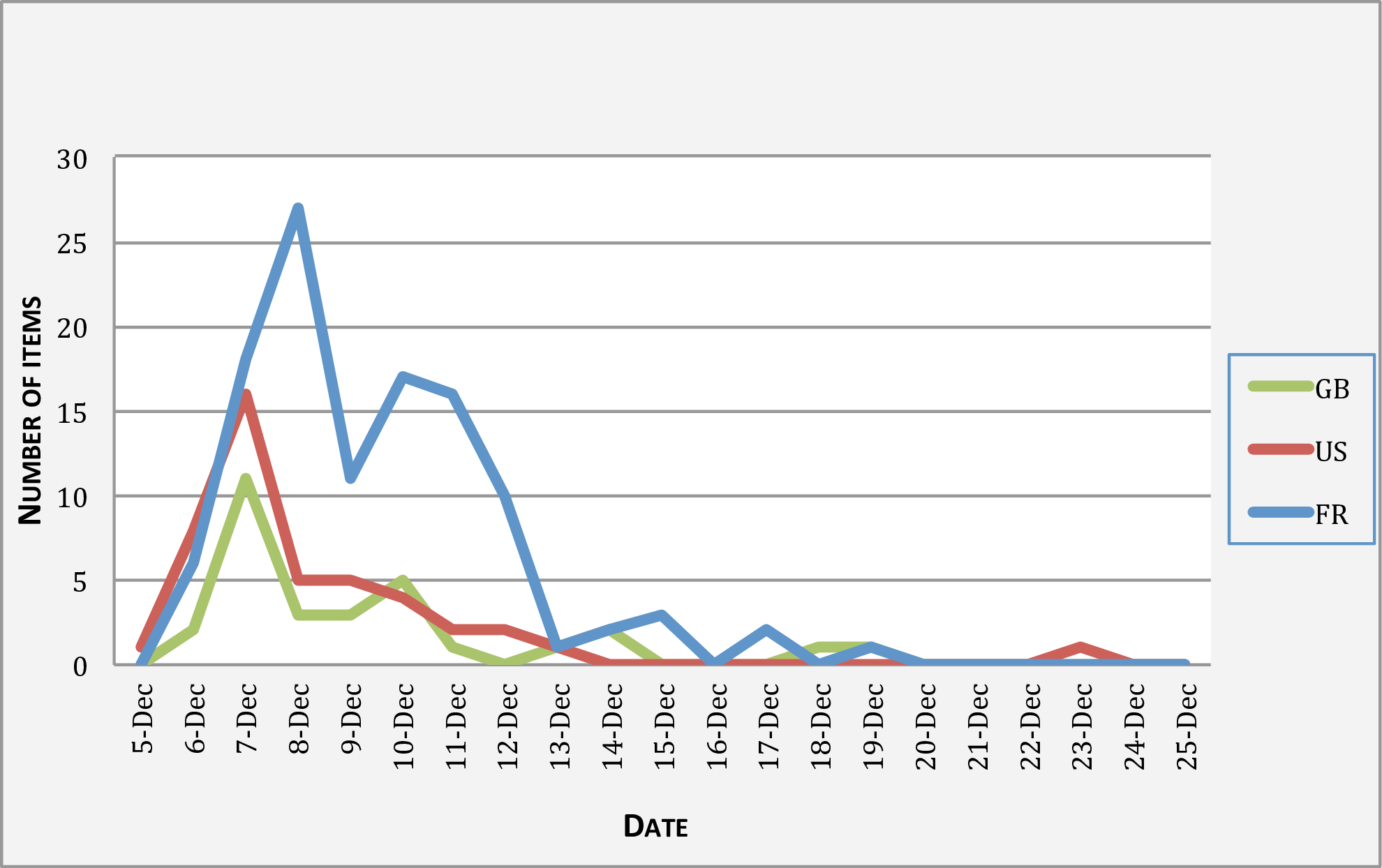

Supplement: S3 Fig — (TIF) [file pone.0158393.s003.tif]

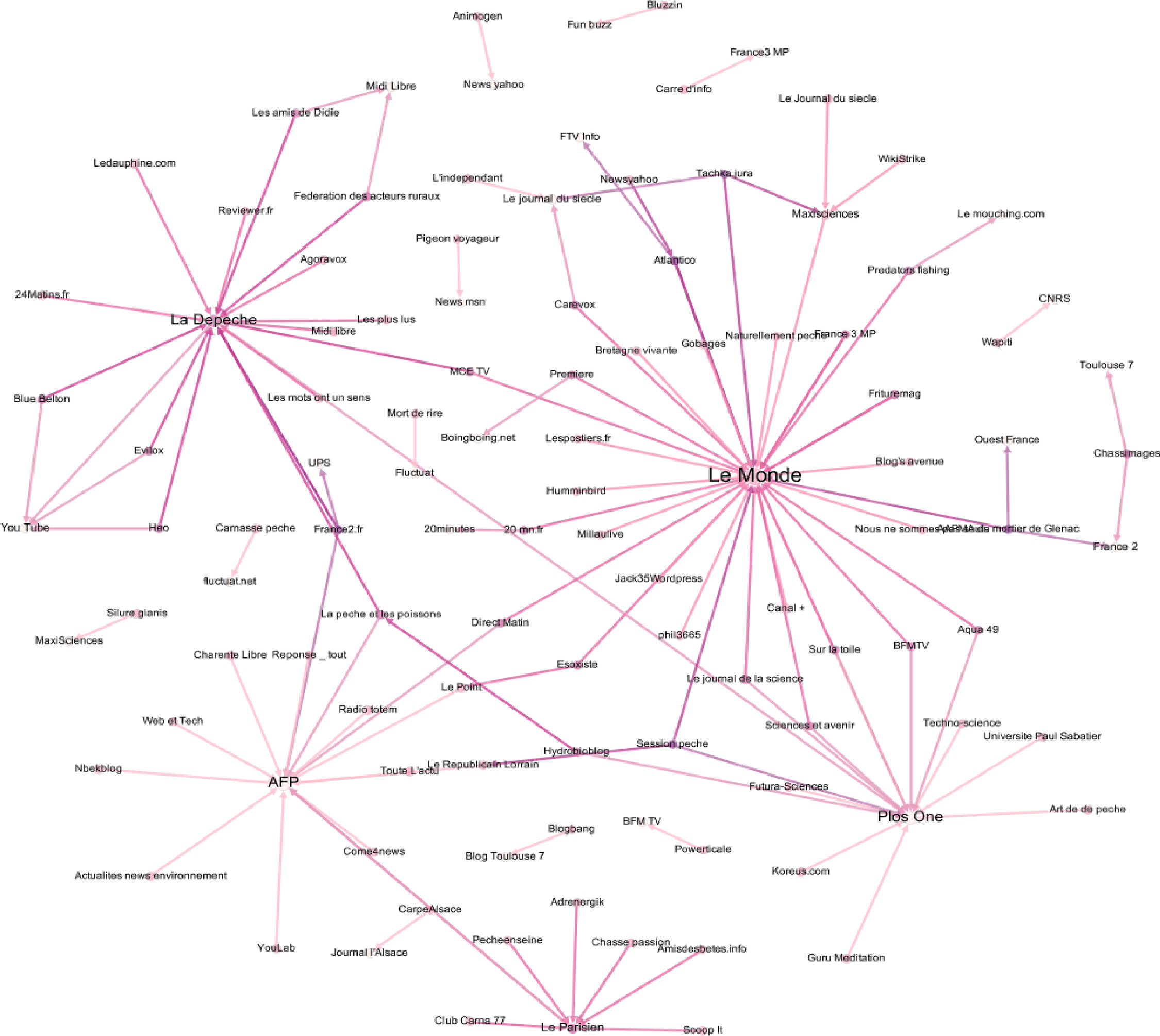

Supplement: S4 Fig — This figure gives a clear account of the complexity of the circulation network of the Plos One article in the Web-based media sphere and highlights one of the relay nodes analysed in France: the scientific blog Passeur de Sciences (Le Monde). (TIF) [file pone.0158393.s004.tif]

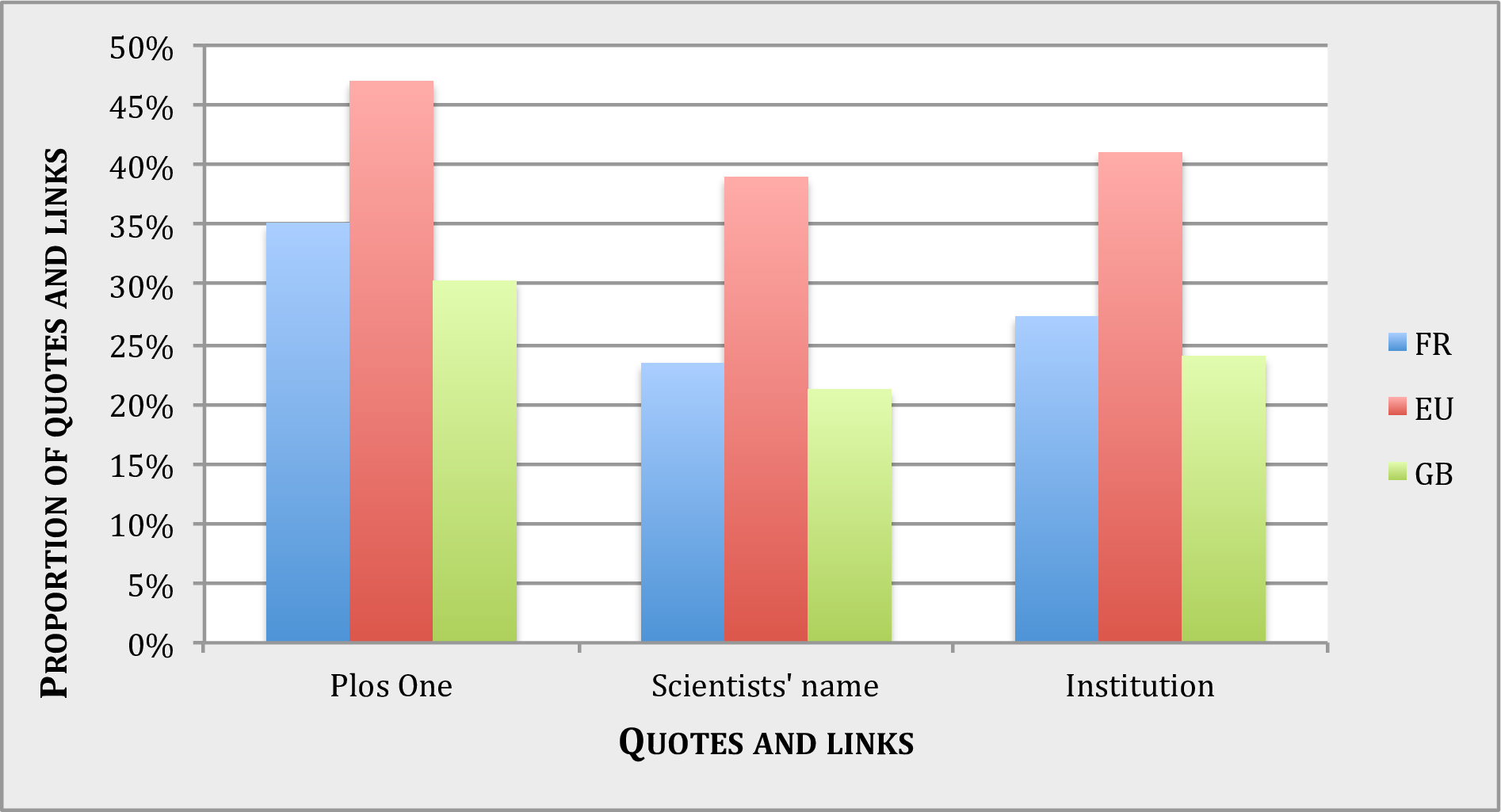

Supplement: S5 Fig — (TIF) [file pone.0158393.s005.tif]

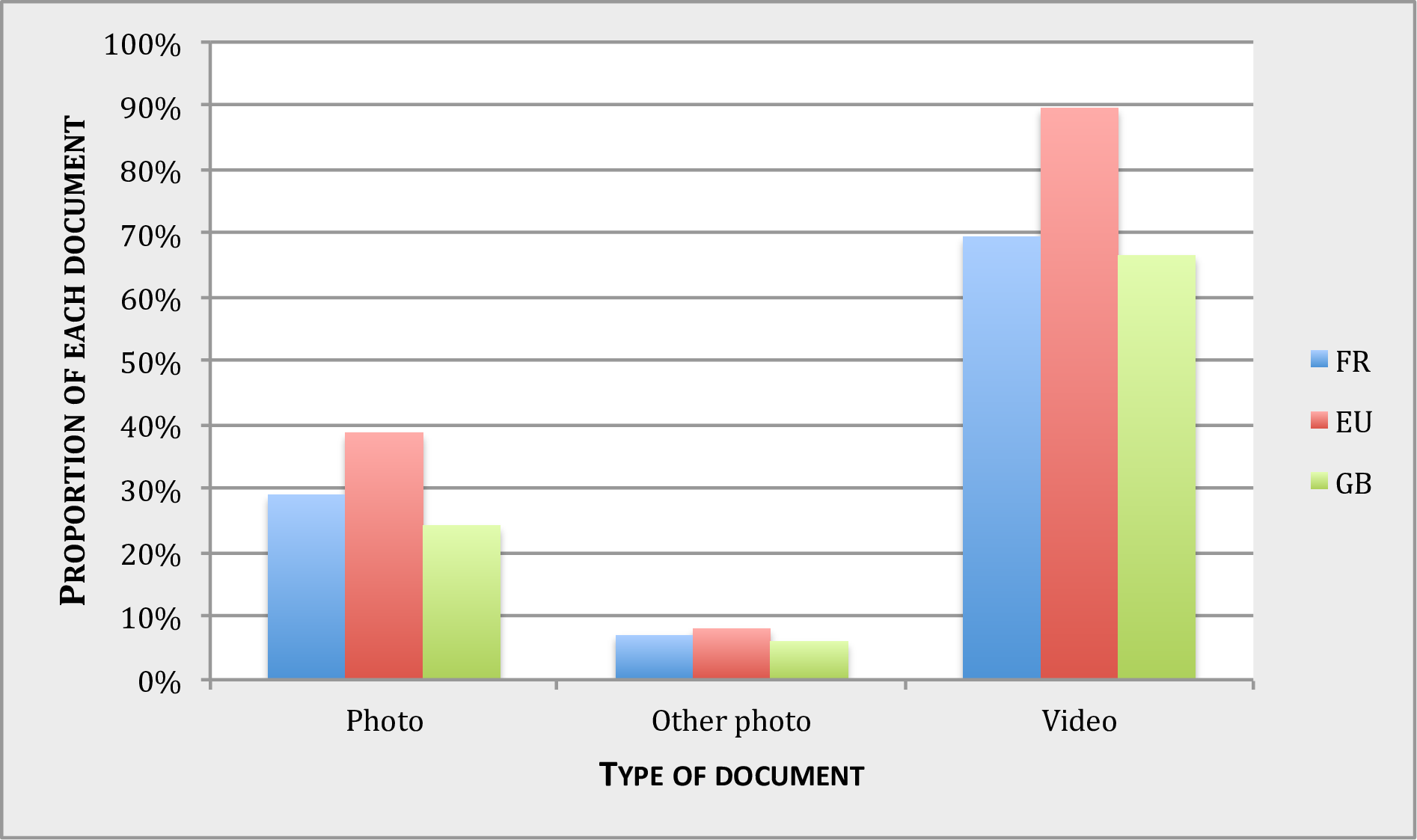

Supplement: S6 Fig — (TIF) [file pone.0158393.s006.tif]

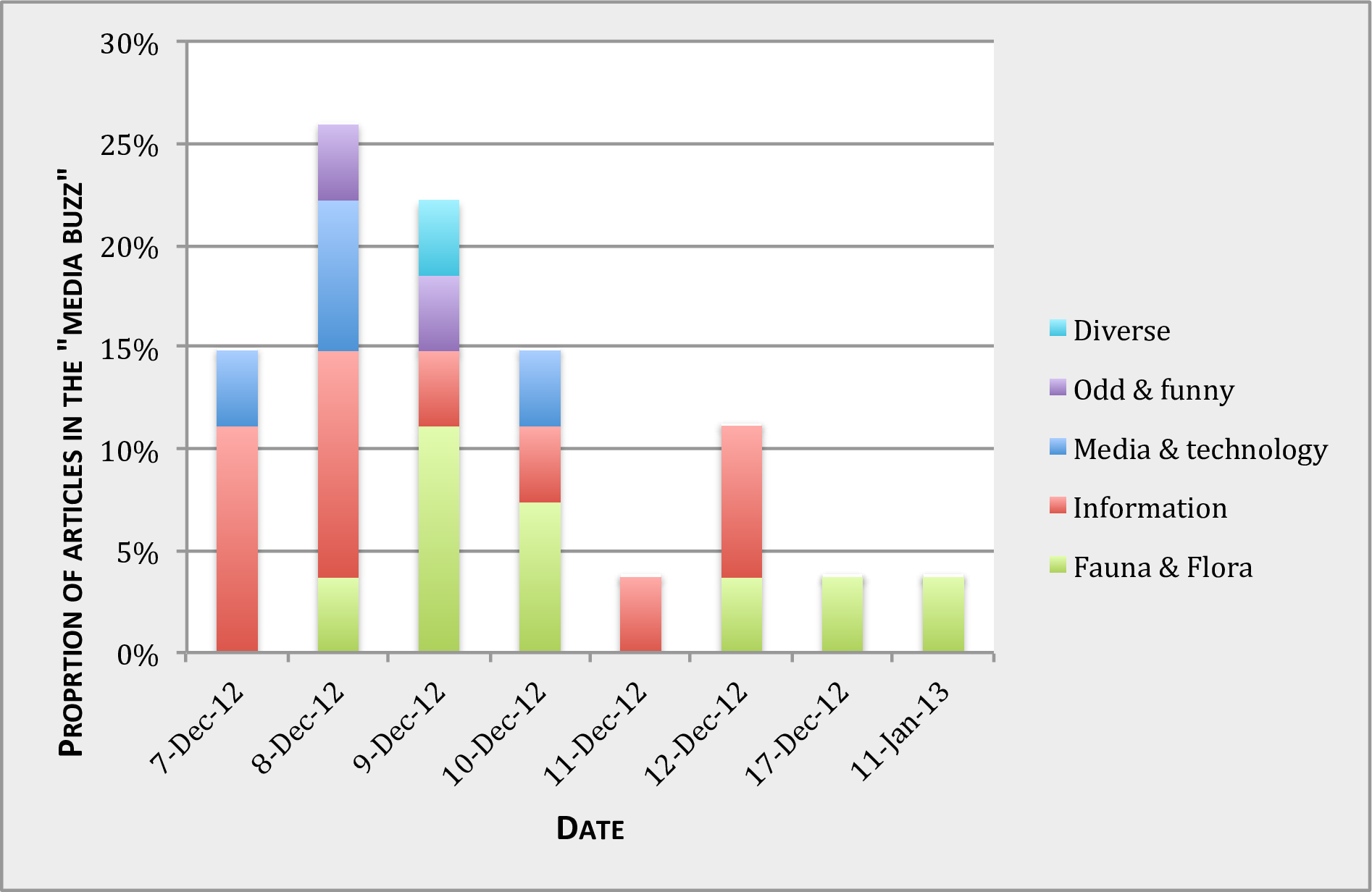

Supplement: S7 Fig — (TIF) [file pone.0158393.s007.tif]
